# Supplementary material for: Genome-Wide Analysis of ATP Binding Cassette (ABC) Transporters in Peach (Prunus persica) and Identification of a Gene PpABCC1 Involved in Anthocyanin Accumulation
Source: Int J Mol Sci. 2023 Jan 18;24(3):1931. doi: 10.3390/ijms24031931 (PMC9916050; doi:10.3390/ijms24031931)
Supplement: Supplementary file 1 [file ijms-24-01931-s001.zip › Supplementary Figure.pptx]

## Slide 1
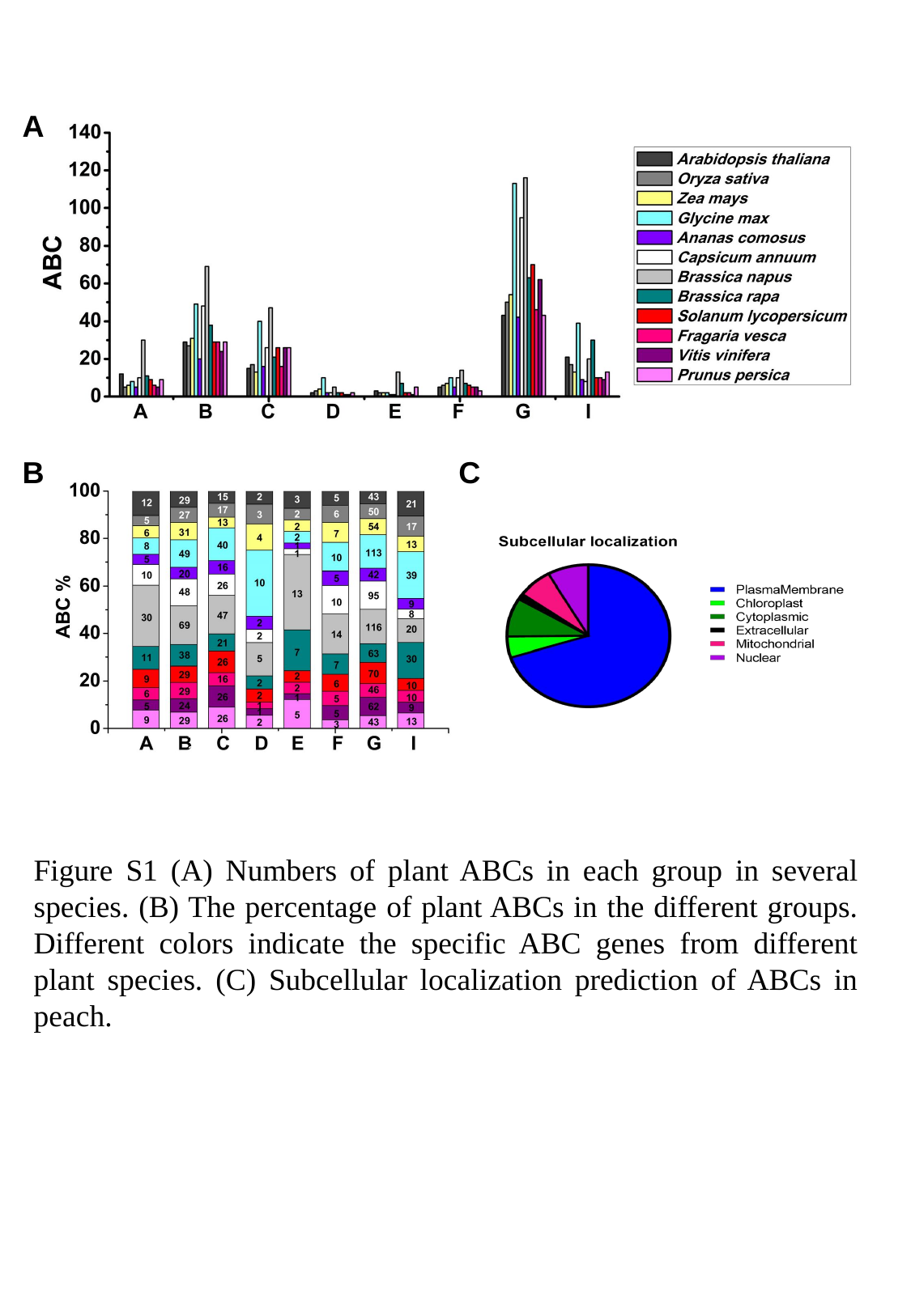

A
B
C
Figure S1 (A) Numbers of plant ABCs in each group in several species. (B) The percentage of plant ABCs in the different groups. Different colors indicate the specific ABC genes from different plant species. (C) Subcellular localization prediction of ABCs in peach.

## Slide 2
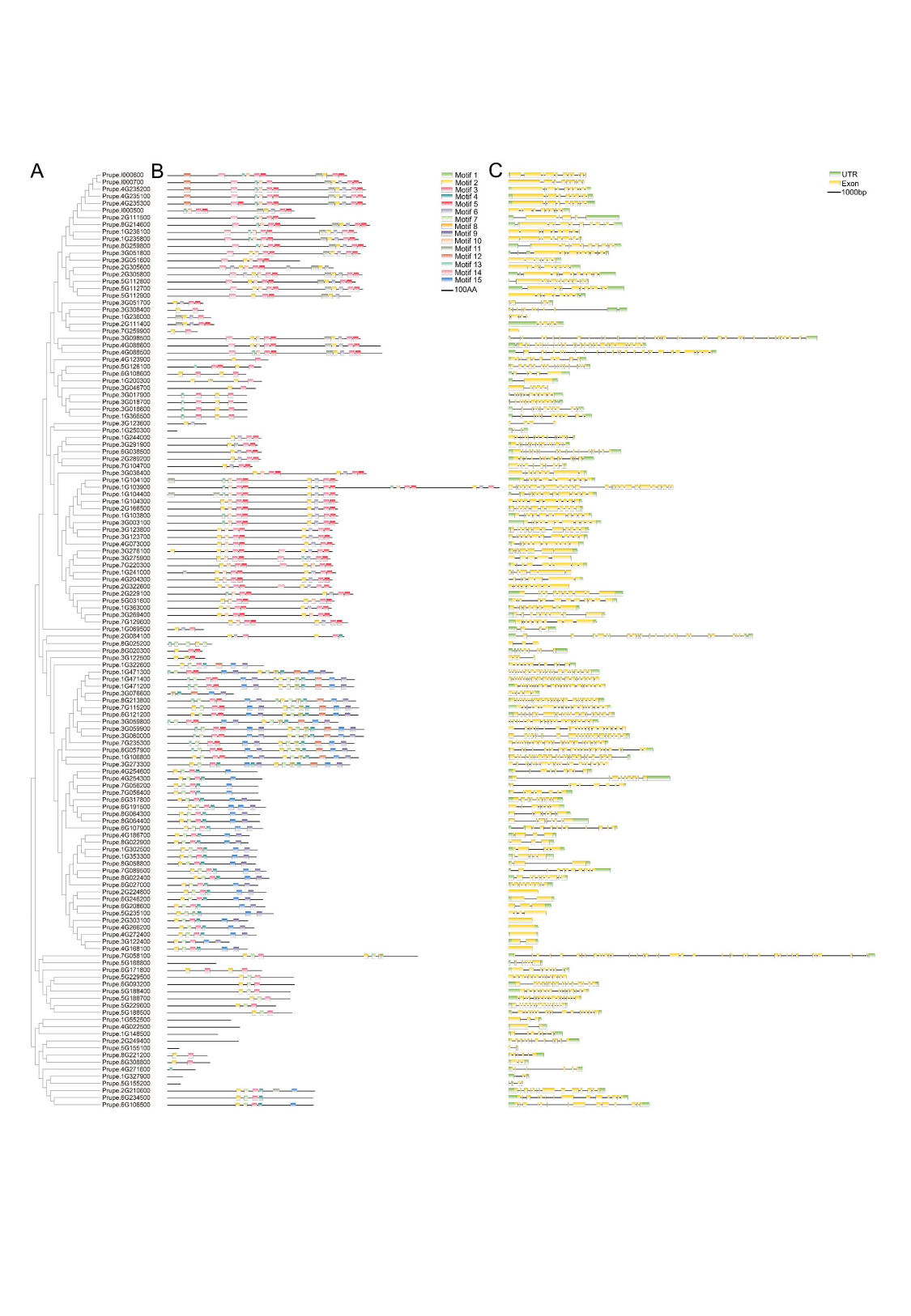

## Slide 3
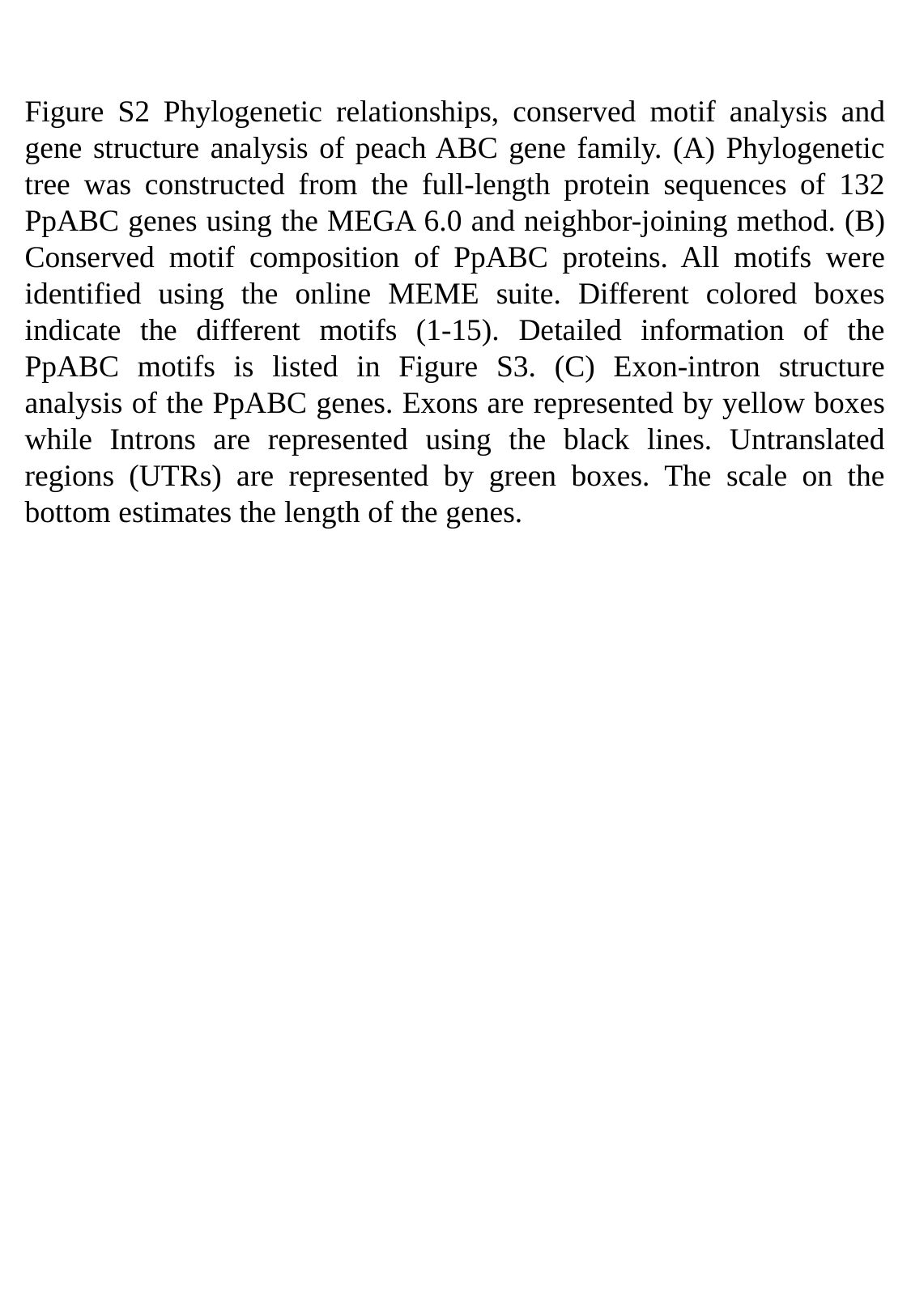

Figure S2 Phylogenetic relationships, conserved motif analysis and gene structure analysis of peach ABC gene family. (A) Phylogenetic tree was constructed from the full-length protein sequences of 132 PpABC genes using the MEGA 6.0 and neighbor-joining method. (B) Conserved motif composition of PpABC proteins. All motifs were identified using the online MEME suite. Different colored boxes indicate the different motifs (1-15). Detailed information of the PpABC motifs is listed in Figure S3. (C) Exon-intron structure analysis of the PpABC genes. Exons are represented by yellow boxes while Introns are represented using the black lines. Untranslated regions (UTRs) are represented by green boxes. The scale on the bottom estimates the length of the genes.

## Slide 4
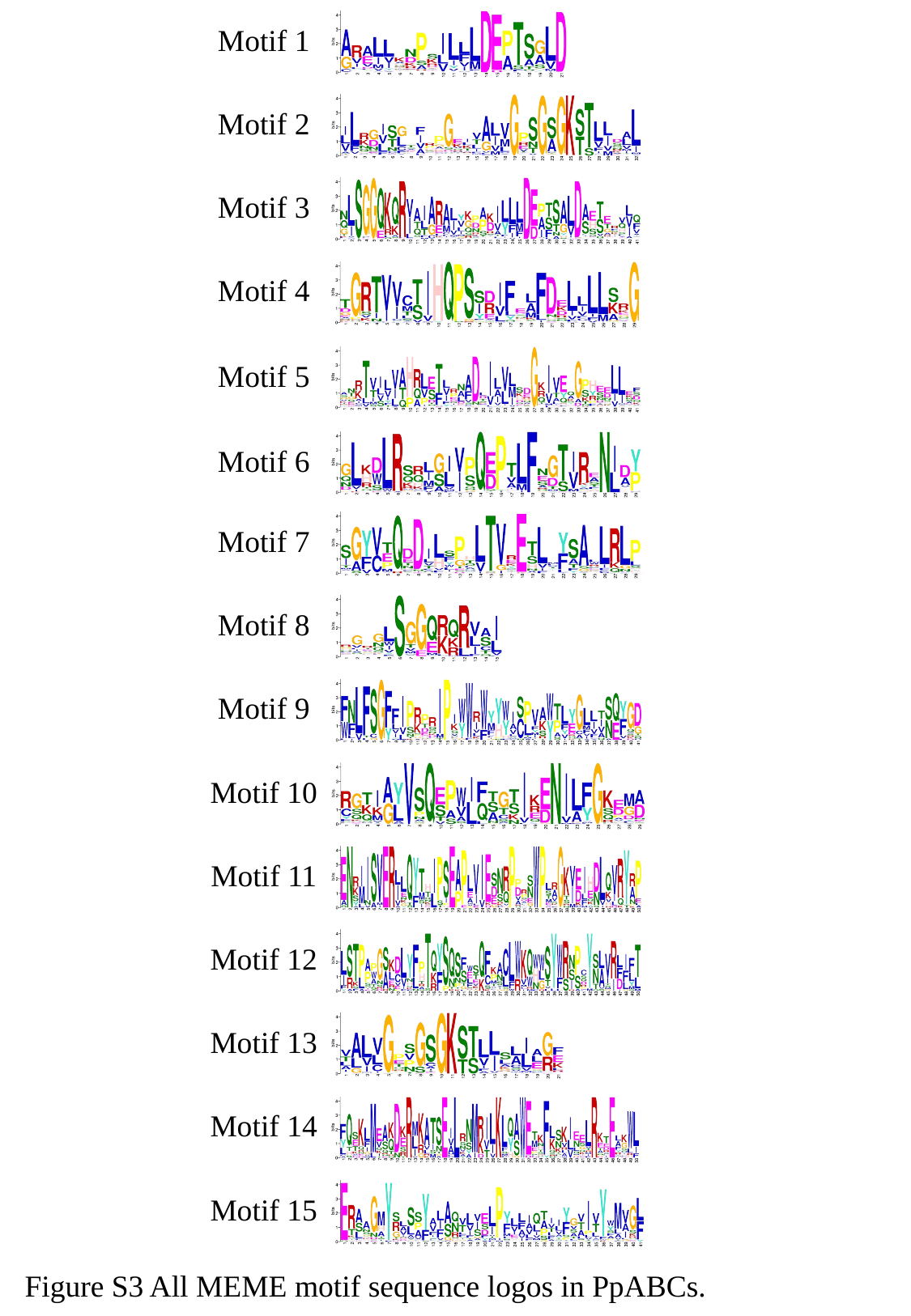

Motif 1
Motif 2
Motif 3
Motif 4
Motif 5
Motif 6
Motif 7
Motif 8
Motif 9
Motif 10
Motif 11
Motif 12
Motif 13
Motif 14
Motif 15
Figure S3 All MEME motif sequence logos in PpABCs.

## Slide 5
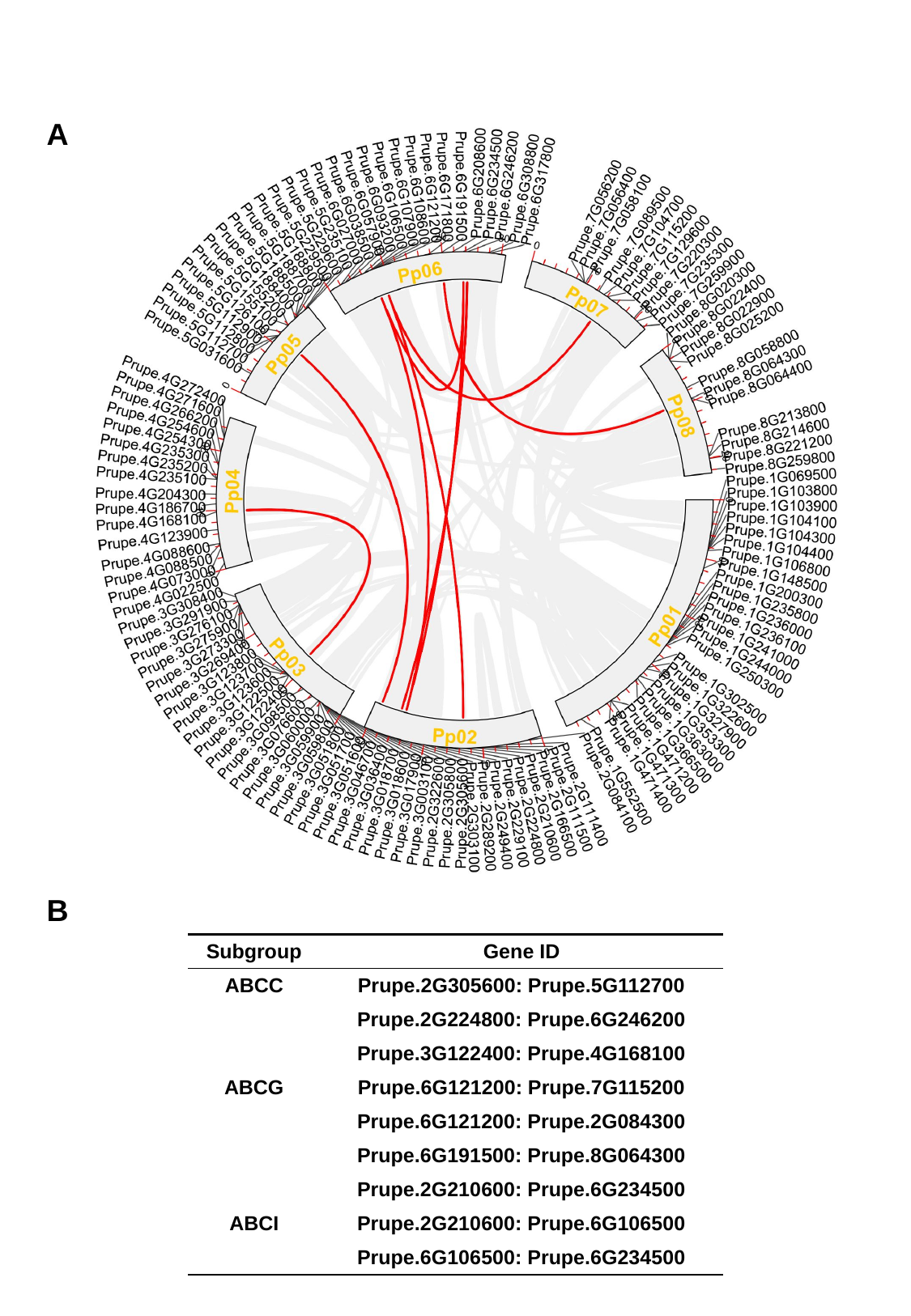

A
B
| Subgroup | Gene ID |
| --- | --- |
| ABCC | Prupe.2G305600: Prupe.5G112700 |
| ABCG | Prupe.2G224800: Prupe.6G246200 |
| | Prupe.3G122400: Prupe.4G168100 |
| | Prupe.6G121200: Prupe.7G115200 |
| | Prupe.6G121200: Prupe.2G084300 |
| | Prupe.6G191500: Prupe.8G064300 |
| ABCI | Prupe.2G210600: Prupe.6G234500 |
| | Prupe.2G210600: Prupe.6G106500 |
| | Prupe.6G106500: Prupe.6G234500 |

## Slide 6
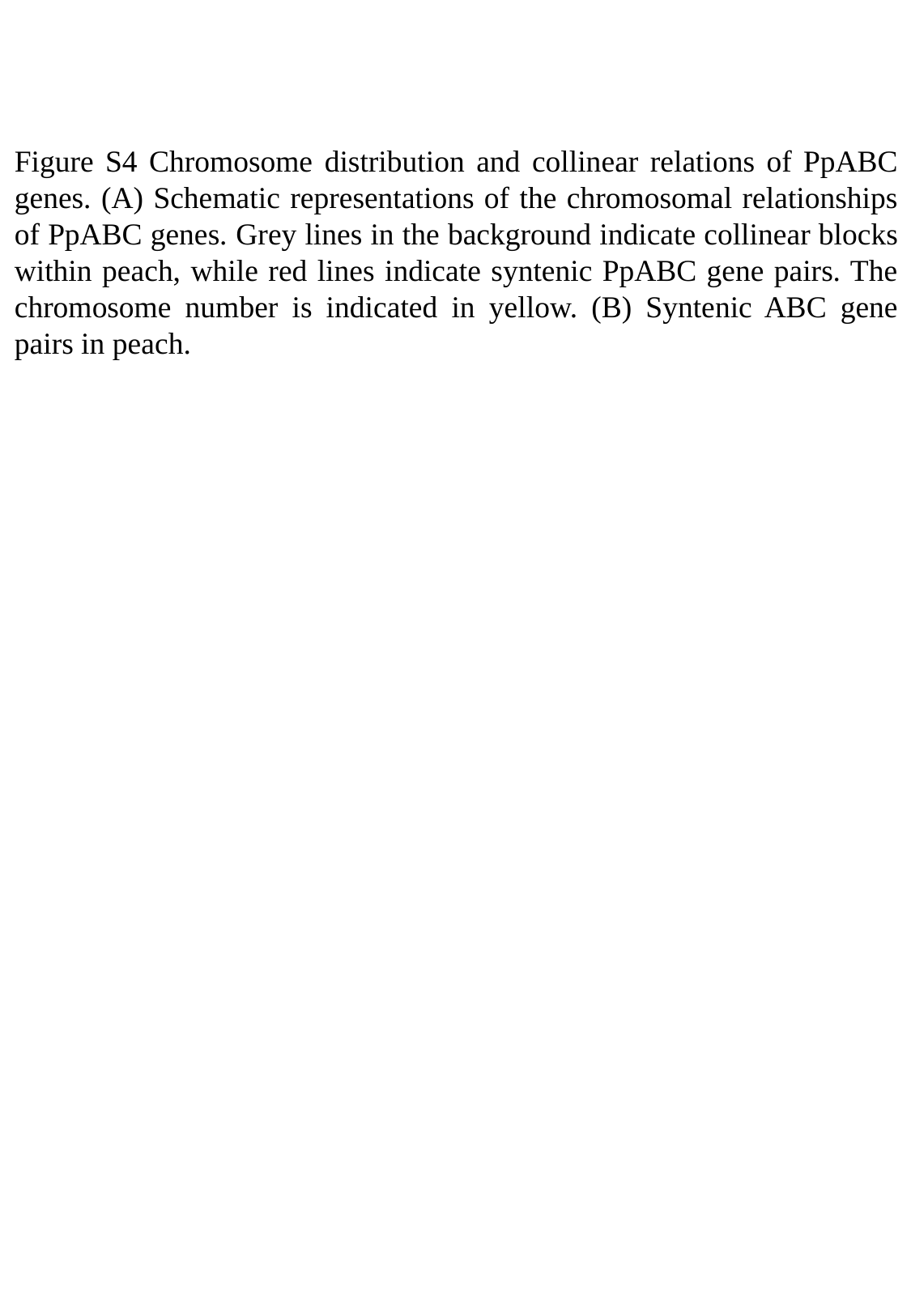

Figure S4 Chromosome distribution and collinear relations of PpABC genes. (A) Schematic representations of the chromosomal relationships of PpABC genes. Grey lines in the background indicate collinear blocks within peach, while red lines indicate syntenic PpABC gene pairs. The chromosome number is indicated in yellow. (B) Syntenic ABC gene pairs in peach.

## Slide 7
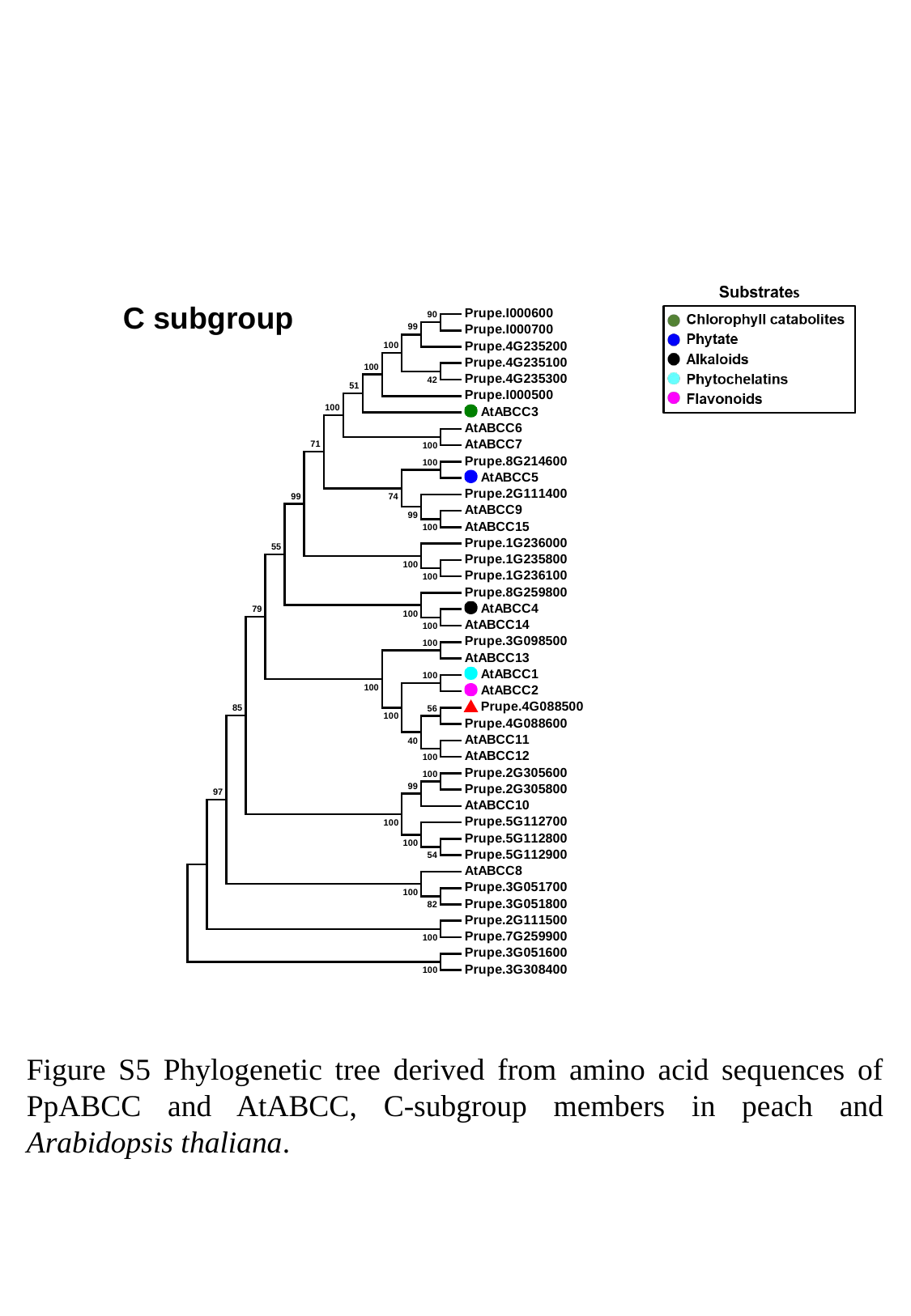

C subgroup
Figure S5 Phylogenetic tree derived from amino acid sequences of PpABCC and AtABCC, C-subgroup members in peach and Arabidopsis thaliana.
